# Une association malformative exceptionnelle: situs inversus totalis et hernie rétro-costo-xyphoidienne

**DOI:** 10.11604/pamj.2019.32.167.6558

**Published:** 2019-04-09

**Authors:** Badr Slioui, Mohammed Abdellaoui, Soufiane Belabbes, Rachida Dafiri

**Affiliations:** 1 Service de Radiologie Pédiatrie, Hôpital d’Enfant, Rabat, Maroc

**Keywords:** Morgagni-Larrey hernia, situs inversus totalis, congenital malformation, Hernie de Larrey-Morgagni, situs inversus totalis, malformations congénitales

## Abstract

La hernie rétro-costo-xyphoïdienne est une malformation congénitale rare. Elle représente 3% de l'ensemble des hernies diaphragmatiques. Elle peut être isolée ou associée à d'autres malformations. Nous rapportons une observation rarissime d'hernie de Larrey-Morgagni et de situs inversus totalis découverts à la suite d'une détresse respiratoire néonatale.

## Abstract (en)

Retro-costo-xyphoidian hernia is a rare congenital malformation. It accounts for 3% of all the diaphragmatic hernias. It can be isolated or associated with other malformations. We here report a very rare case of Morgagni-Larrey hernia and situs inversus totalis detected after neonatal respiratory distress.

## Introduction

La hernie rétro-costo-xyphoïdienne est une malformation congénitale rare. Elle représente 3% de l'ensemble des hernies diaphragmatiques [1]. Le diagnostic de cette malformation est souvent retardé car la maladie reste généralement asymptomatique. Parfois, la hernie peut se manifester par des signes digestifs ou respiratoires. Rarement c'est à l'occasion d'un traumatisme ou lors de la survenue d'une complication que la hernie est découverte. La hernie rétro-costo-xyphoïdienne peut être soit isolée soit associée à d'autres malformations (70% des cas): cardiaques (25%), chromosomiques (15%), rénales, digestives et squelettiques. L'association d'une hernie rétro-costo-xyphoïdienne et d'un situs inversus totalis reste exceptionnelle. A notre connaissance, c'est le premier cas décrit dans la littérature. Le situs inversus totalis est une maladie autosomique récessive rare (2 pour 10.000 naissances vivantes) dans laquelle l'orientation de tous les organes asymétriques dans le corps est une image miroir de la morphologie normale [2-6]. Nous rapportons une observation rarissime d'hernie de Larrey-Morgagni et de situs inversus totalis découverts à la suite d'une détresse respiratoire néonatale.

## Patient et observation

**Figure 1:**
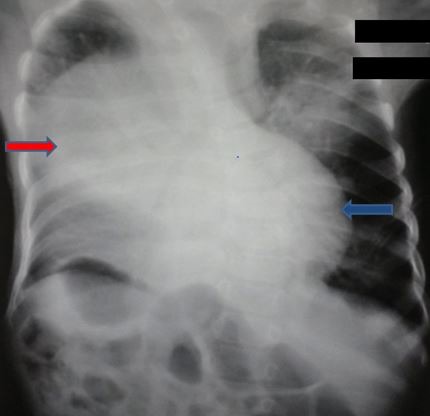
radiographie de thorax face: opacité médiastinale antérieure (flèche rouge) effaçant les bords du cœur (flèche bleu), élargissant le médiastin moyen et inférieur

**Figure 2:**
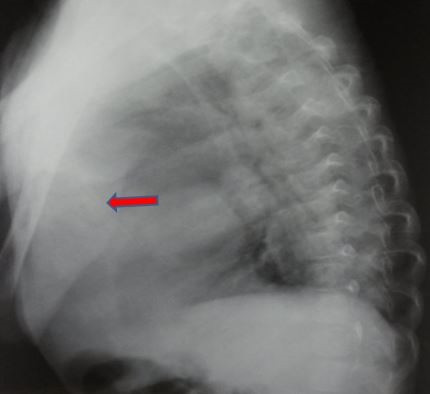
radiographie de thorax profil: confirme le siège antérieur de l’opacité (flèche rouge)

**Figure 3:**
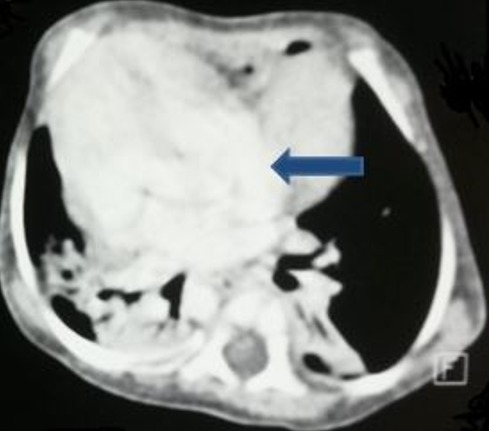
TDM en coupes axiales C+: A) cavités cardiaques comprimées par la hernie (flèche bleu); B) hernie intra-thoracique de la quasi-totalité du foie (flèche rouge)

**Figure 4:**
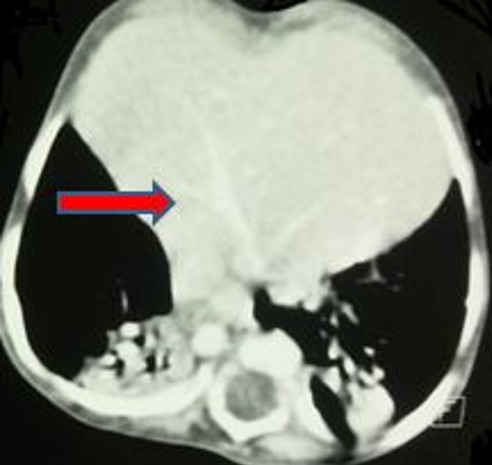
TDM en coupes axiales C+: localisation en miroir du cœur et de la rate (flèche orange)

**Figure 5:**
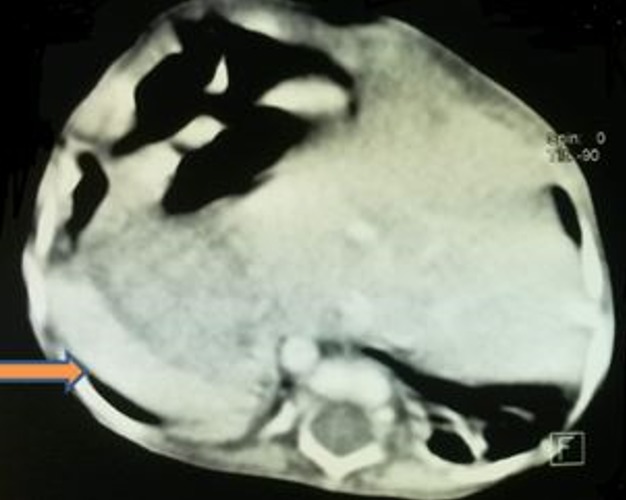
TDM en reconstruction coronale C+: issue du foie en intra-thoracique (flèche verte)

**Figure 6:**
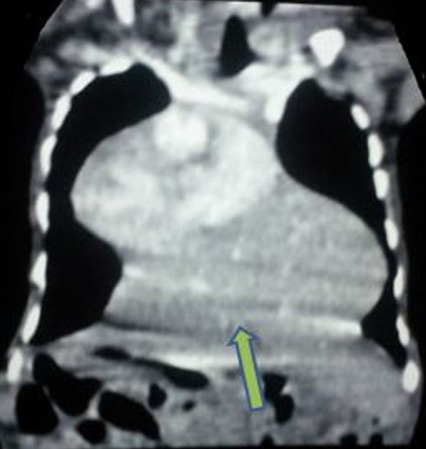
TDM en reconstruction sagittale C+: volumineuse solution de continuité diaphragmatique rétro-costo-xiphoïdienne (flèche verte) avec issue du foie en intra-thoracique

Il s'agit d'un nouveau-né de sexe masculin, de parents consanguins, issue d'une grossesse mal suivie. L'accouchement s'est déroulé par voie basse après 39 semaines de gestation. A la naissance, le score d'Apgar est à 9/10 à 5 min, le poids à 3000g et le périmètre crânien à 32 cm. A 3 semaines de vie, le nouveau-né présente un tableau de détresse respiratoire avec tachypnée, respiration paradoxale, cyanose et signes de lutte. La radiographie thoracique réalisée en urgence (Figure 1 et Figure 2) objective une volumineuse opacité homogène médiastinale latéralisée à droite, effaçant le bord cardiaque droit, comblant l'angle cardiophrénique homolatéral. Le complément scannographique (Figure 3, Figure 4, Figure 5, Figure 6) montre une solution de continuité diaphragmatique rétro-costo-xyphoïdienne avec hernie intra-thoracique de la quasi-totalité du foie et compression des cavités cardiaques qui sont refoulées en haut. La tomodensitométrie (TDM) révèle également une localisation en miroir du cœur, de la crosse aortique et de la rate. Le diagnostic d'un situs inversus totalis avec hernie de Larrey-Morgagni compressive a été posé. Après stabilisation, le nourrisson a été opéré après 10 jours de séjour en réanimation. L'intervention par laparotomie médiane a consisté en une résection du sac herniaire, une réintégration du foie dans la cavité abdominale et une suture diaphragmatique. L'évolution a été marquée par une amélioration des signes respiratoires.

## Discussion

Les hernies diaphragmatiques congénitales antérieures sont rares. Elles représentent 3% des hernies congénitales diaphragmatiques [1]. Il s'agit d'une ascension d'un ou plusieurs organes abdominaux en intra-thoracique à travers les foramens de Larrey ou de Morgani [5, 6]. Les manifestations cliniques des hernies rétro-costo-xyphoïdiennes sont variables. Le diagnostic est habituellement tardif, souvent fortuit lors de la réalisation d'une radiographie du thorax pour une autre pathologie [5, 7, 8]. Parfois, la hernie est symptomatique notamment chez les nourrissons et les enfants, occasionnant des troubles digestifs ou respiratoires tels une dyspnée ou des infections pulmonaires à répétition [7]. La survenue d'une détresse respiratoire comme le cas de notre observation reste une présentation clinique atypique. Dans certains cas, c'est à l'occasion d'un traumatisme ou d'une complication notamment occlusive que la maladie est révélée. Il existe 2 théories pour expliquer la survenue d'une hernie rétro-costo-xyphoïdienne [9, 10]. La théorie congénitale est acquise. La première hypothèse est admise par la majorité des auteurs. Elle est confortée par l'association fréquente de ce type d'hernie à d'autres malformations. En effet les anomalies associées sont retrouvées dans 70% des cas [1]. Il peut s'agir de cardiopathies congénitales dans 25% des cas, de malrotation digestive dans 20% des cas, de trisomie 21 dans 15% des cas. Plus rarement, on a rapporté l'association avec une tétralogie de Fallot, une omphalocèle, un hypospadias, une hernie ombilicale, des anomalies vertébrales ou thoraciques (pectusexcavatus, carinatus) [9]. Les hernies rétro-costo-xyphoïdiennes pourraient également faire partie du syndrome de Cantrell ou de celui de Williams et Beuren [11]. L'association d'une hernie diaphragmatique et un situs inversus totalis reste exceptionnel. Un seul cas a été rapporté dans la littérature anglaise [12]. Il s'agissait d'une hernie de Bochdaleck. L'association d'un situs inversus totalis avec une hernie de type Larrey-Morgagni n'a jamais été décrite à notre connaissance. Le diagnostic d'une hernie de Morgagni ou de Larrey est évoqué à la radiographie thoracique qui montre une opacité médiastinale antérieure et inférieure, de densité variable selon le contenu des organes herniaires. La confirmation est scannographique objectivant une ascension des organes abdominaux en intra-thoracique à travers un defect diaphragmatique antérieur [13]. L'imagerie par résonance magnétique (IRM) peut être utile en cas de doute pour identifier le defect dans la paroi diaphragmatique [13]. Le traitement de la hernie rétro-costo-xyphoïdienne est chirurgical [7, 14]. La voie d'abord abdominale est la plus utilisée soit par une laparotomie ou une laparoscopie. La fermeture de l'orifice ne pose souvent aucun problème. L'indication opératoire chez les sujets symptomatiques est formelle. En cas de découverte fortuite chez des nouveau-nés ou des enfants jeunes, vu les risques anesthésiques et la difficulté de réaliser un acte mini-invasive, une chirurgie préventive pose une problématique de délai [1, 10, 15, 16].

## Conclusion

La hernie rétro-costo-xyphoïdienne est une entité rare, de découverte le plus souvent fortuite. Néanmoins, cette hernie peut occasionner chez l'enfant et le nourrisson une détresse respiratoire. Le diagnostic est évoqué sur la radiographie standard et confirmé par le scanner qui permet le diagnostic positif, la détection des complications ainsi que le dépistage des malformations associées.

## Conflits d’intérêts

Les auteurs ne déclarent aucun conflit d'intérêts.
